# Identification and Expression Analysis of Ras Gene in Silkworm, *Bombyx mori*

**DOI:** 10.1371/journal.pone.0008030

**Published:** 2009-11-25

**Authors:** Takehiko Ogura, Anjiang Tan, Takuya Tsubota, Takayo Nakakura, Takahiro Shiotsuki

**Affiliations:** 1 Department of Applied Life Sciences, Kyoto University, Kyoto, Japan; 2 Invertebrate Gene Function Research Unit, National Institute of Agrobiological Science, Tsukuba, Ibaraki, Japan; University of Texas MD Anderson Cancer Center, United States of America

## Abstract

Ras proteins play important roles in development especially for cell proliferation and differentiation in various organisms. However, their functions in the most insect species are still not clear. We identified three *ras* cDNAs from the silk worm, *Bombyx mori*. These sequences corresponded to three Ras of *Drosophila melanogaster*, but not to three mammalian Ras (H-Ras, K-Ras, N-Ras). Subsequently, the expression profiles of *ras* were investigated by quantitative real-time PCR using whole body of individuals from the embryonic to adult stages, and various tissues of 4th and 5th instar larvae. Each of three *Bombyx ras* showed different expression patterns. We also showed membrane localization of their products. These results indicate that the three *Bombyx* Ras are functional and have different roles.

## Introduction

The *ras* genes was first isolated as the oncogene [1]–[3], and it was shown that its product Ras possesses GTPase activity [4]. Ras and structurally related proteins form the small GTPase family, a superfamily of monomeric GTP binding proteins (G-protein), which exists in various organisms [5], [6]. Numbers of studies of small GTPase have been reported in relation to their characteristics and distribution to variety of organisms [structures [7]–[9], functions [10], [11] and oncogenesis [12]–[15] of mammals [16]–[18], plants [19] and fungi [20]–[24]]. Commonly, the small GTPase plays central roles in signal transduction across membranes via the interaction with GDP/GTP exchange factors (GEF), GTPase activation proteins (GAP) and other regulators [10], [25]–[27]. Among small GTPases, Ras subfamily members work with certain membrane receptors such as receptor tyrosine kinases (RTK) [28]–[30]. When these receptors accept extracellular signaling factors such as growth factors and hormones, signaling cascades start via the phosphorylation of proteins. Ras acts as a switch in the middle of such signaling cascades to regulate the downstream MAPK (mitogen-activated protein kinase) signaling pathway [28], [31], [32]. It is well known that the Ras/MAPK pathway exists in various signaling cassettes, and is crucially involved in the proliferation of a cell and the control of cell differentiation [33]–[35].

Invertebrates also possess Ras proteins [36]–[42]. Among invertebrates, a nematode *Caenorhabditis elegans* and the fruit fly *Drosophila melanogaster* have been used concentrically in various studies to elucidate the function of Ras, since they are appropriate for research using mutants. In *C. elegans*, various defective phenotypes during the development, which were casused by Ras pathway mutations, led the discovery of basic components and the framework of the RTK/Ras/MAPK signaling pathway [43]. Also in *D. melanogaster*, mutants related to Ras were studied and provided basic information on the function of Ras [44], [45], after the identification of three Ras homologues [40]–[42], [46]–[48]. Such studies revealed the importance of epithelial growth factor receptors (EGFR: one goup of RTK) and their downstream Ras pathways for various developmental events of *Drosophila* ranging from cell fate determination during embryogenesis to the control of cell apoptosis [45]. Imaginal disc proliferation and appendage differentiation during larvae/pupae stages [44], [49]–[51], cell fate determination during embryogenesis [52], polarization of body axes during oogensis [53] and the control of cell apoptosis [54] were intensively studied for Ras. Thus, we can acquire a lot of information on *D. melanogaster* Ras. On the other hand, in relation to Ras of other insect species, there are few reports of the characteristics and the mode of action. Furthermore, *ras* cDNA sequences of insects except for *D. melanogaster* have not been reported, although several cDNA sequences have been predicted from the genome data of some insect species.

During the growth of insects, the timing of developmental events, such as molting and metamorphosis, is strictly regulated by two peripheral hormones, juvenile hormone (JH) and 20-hydroxyecdysone (20E) [55]. Recently, in relation to the body size determination of *D. melanogaster*, it was indicated that the secretion of ecdysone, the precursor of 20E, was regulated by the Ras signaling pathway [56]. Similarly, functional links between ecdysone and small GTPases during the development of *Drosohila* have begun to be reported [57]–[59]. These reports suggest that the modes of action of 20E, and probably JH, are closely associated with the Ras function during insect development. However, such relations shown so far are indirect, and the precise mode of such interactions is ambiguous. It is well known that the effects of peripheral hormones, especially the roles of 20E in the metamorphosis and function of JH, are not clear in *Drosophila*. Furthermore, precise roles of Ras of *D. melanogaster* and other insects are expected to be considerably different, since the patterns of the development and body structures are highly diverse among insect species. Therefore, Ras studies using various insect species are thought to provide more beneficial information on the direct link between the hormonal regulation of insect growth and the function of Ras in the molecular interaction level.

The silkworm *Bombyx mori* is an insect which is frequently used for endocrinological research, and is also suitable for genetic research. In addition, the effect of JH is very clear in *B. mori* compared with *D. melanogaster*. Thus, *B. mori* is advantageous species to study the interaction between the hormonal regulation of insect growth and the function of Ras. Moreover, the genome database, KAIKOBASE (http://spg.dna.affrc.go.jp/KAIKObase/), which have been established recently, will promote such studies efficiently. In this study, to elucidate the existence of the Ras/MAPK pathway in *B. mori* and its contribution to growth, we identified three *ras* genes of *B. mori* (*Bmras*: *Bmras1*, *Bmras2* and *Bmras3*) and determined their cDNA sequences. Furthermore, we precisely investigated the developmental expression profiles of *Bmras* by quantitative real-time PCR in various tissues. The results of this study suggest different roles of the three *Bombyx* Ras (BmRas: BmRas1, BmRas2 and BmRas3) during development.

## Results

### cDNA Cloning and Sequence Analysis of *Bmras*

**Figure 1:**
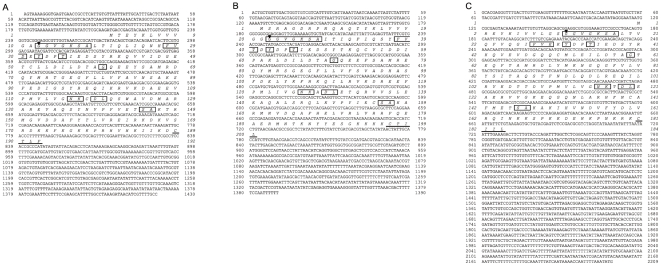
cDNA and putative amino acid sequences of B. mori Ras. (A) Ras1 (accession number: AB3892674), (B) Ras2 (AB3892674) and (C) Ras3 (AB3892674). Amino acids which are important for GTP/Mg^2+^ binding are boxed. The C terminal isoprenylation site is underlined. Glycine residues substituted by valine in BmRas-GFP fusion proteins are indicated by arrowheads.

**Figure 2:**
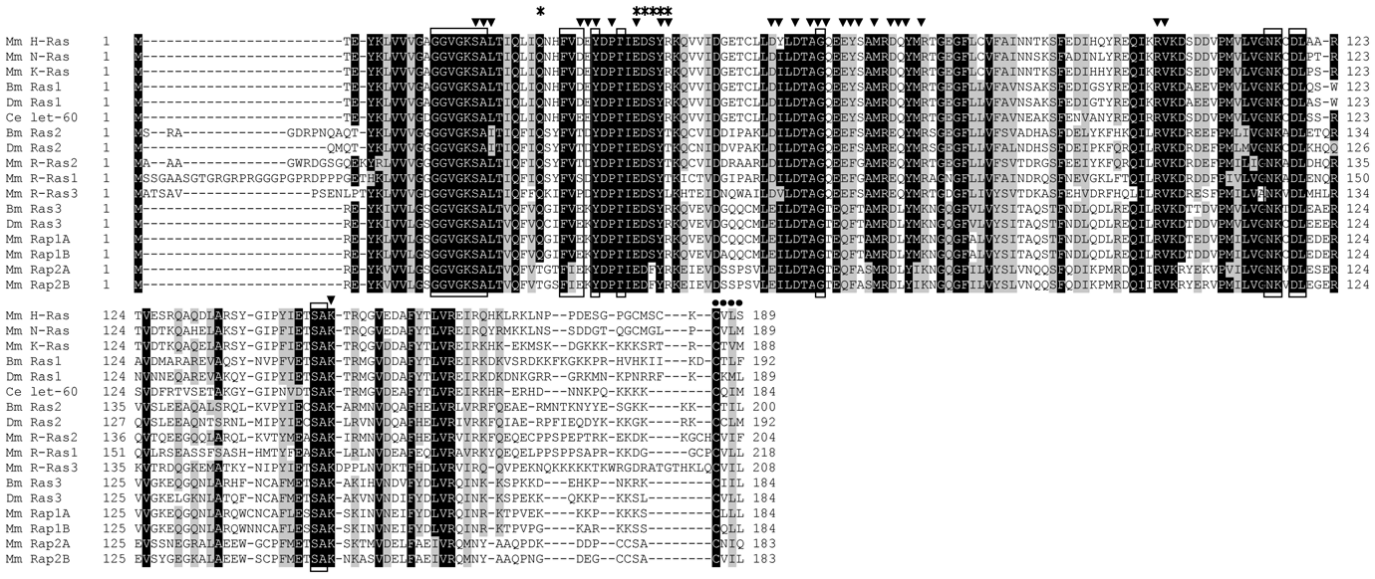
Alignment of amino acid sequences of Ras superfamily members. Bm, Ce, Dm and Mm means *B. mori*, *C. elegans*, *D. melanogaster* and *M. musculus*, respectively. The accession number of each sequence is described in Results. Identical and homologous amino acid residues are highlighted and shaded, respectively. Amino acids which are important for GTP/Mg^2+^ binding are boxed. Amino acids of the GEF interaction site and the effector binding site are shown by arrowheads and asterisks, respectively. The C terminal isoprenylation site is indicated with dots.

**Figure 3:**
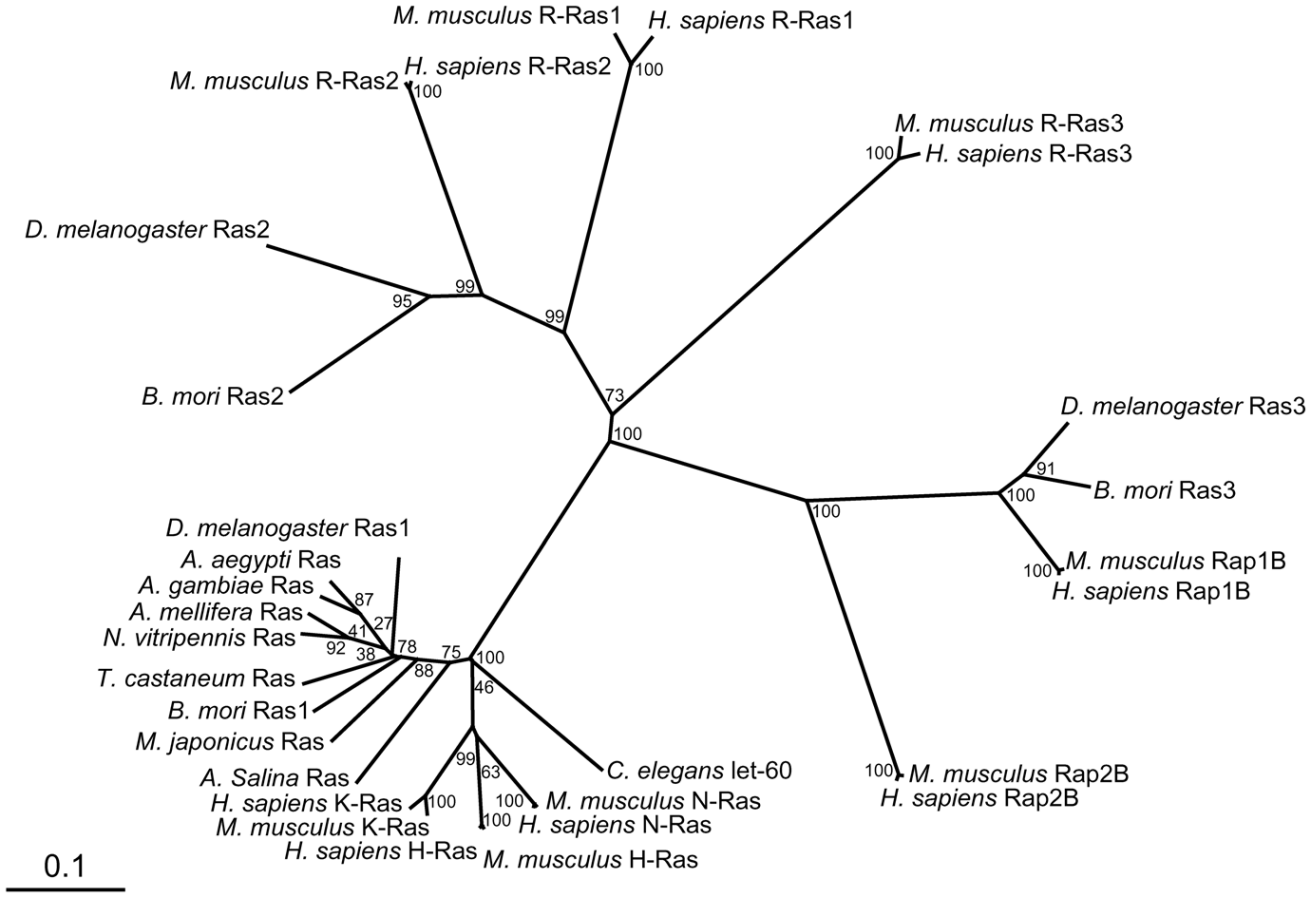
Phylogenic tree constructed using primary sequences of Ras superfamily members. An unrooted UPGMA tree was prepared using CLC FREE WORKBENCH VER. 4.01 (CLC Bio A/S, Aarhus, Denmark). References for sequences are shown in Results. A bootstrap value is attached to each node, and the value is a measure of the confidence in the branch. The number of replicates in bootstrap analysis is adjusted to 100.

A 192 amino acid sequence was deduced from a 1430-bp cDNA sequence, which was determined from PCR products amplified using *ras1* degenerate primers (Table 1). A homology search using BLAST (http://blast.ncbi.nlm.nih.gov/) revealed that this amino acid sequence is highly homologous to that of *D. melanogaster* Ras1. Thus, we determined this sequence as Ras1 of *B. mori* (BmRas1, accession number: AB176555, Fig. 1A). In the same way, 200 and 184 amino acid sequences were deduced from 1390-bp and 2209-bp cDNA sequences, and determined to be Ras2 and Ras3 of *B. mori* (BmRas2, accession number: AB206960, Fig. 1B and BmRas3, accession number: AB170011, Fig. 1C), respectively, from the homology with *D. melanogaster* Ras2 and Ras3. Amino acid sequences of three BmRas were compared with those of *Homo sapiens* H-Ras, K-Ras2b, N-Ras, R-Ras1, R-Ras2, R-Ras3, Rap1B, Rap2B (accession numbers are NP005334, NP004976, NP002515, NP006261, NP036382, NP036351, NP056461 and NP002877, respectivly), *Mus musculus* H-Ras, K-Ras, N-Ras, R-Ras1, R-Ras2, R-Ras3, Rap1A, Rap1B, Rap2A, Rap2B (NP032310, NP067259, NP035067, NP033127, NP080122, NP032650, NP663516, NP077777, NP083795, NP082988), *C. elegans* let-60 (NP502213), *Macrophthalmus japonicus* Ras (AAK14389), *Artemia salina* Ras (P18262), *D. melanogaster* Ras1, Ras2, Ras3 (NP476699, NP523917, NP476857), *Aedes aegypti* Ras (XP001662234), *Anopheles gambiae* Ras (XP307965), *Apis mellifera* Ras (XP394288), *Nasonia vitripennis* Ras (XP001606521) and *Tribolium castaneum* Ras (XP974600). The results are shown in Figs. 2 and 3. Amino acid residues which are conserved and important for small GTPase activity, namely binding with GTP, GEF and effectors, are available from a database (http://blast.ncbi.nlm.nih.gov/Blast.cgi). The alignment of Ras sequences showed that all such residues are conserved in BmRas, especially in relation to residues for binding to GTP (Fig. 2), as in the case of other Ras superfamily members. However, in other parts, their sequences were relatively diverse. C terminal isoprenylation sites (CaaX motif) were also diverse. In phylogenetic analysis, BmRas1, 2 and 3 were the most homologous to *D. melanogaster* Ras1, 2 and 3 (DmRas1, 2 and 3), respectively (Fig. 3). BmRas2 and Bm Ras3 were more homologous to human R-Ras and Rap1, respectively, than three members of the ‘authentic’ Ras subfamily, H-, K- and N-Ras. BmRas1 and DmRas1 comprised a cluster including mammalian H-, K-, N-Ras, *C. elegans* let-60 and Ras of invertebrates with predicted Ras sequences from genome data of other insects. Their relationships in the phylogenetic tree were consistent with taxonomic relationships.

**Table 1:** Primer sequences.

(A)					
Ras1	ras1F	5′-GTRGTYATWGATGGSGARACGTGYYGT-3′			
	ras1R	5′-CGRATYTCRCGCACYARCGTGTAGAASGC-3′			
Ras2	ras2F	5′-TACAARYTNGTNGTNGTNGG-3′			
	ras2R	5′-ARRCTRCAYTTRTTNCCNAC-3′			
Ras3	ras3F	5′-GARAARTAYGAYCCVACSATMGARGA-3′			
	ras3R	5′-TTKATCTGYCKGACCARGTCRTARAA-3′			
(B)					
5′-RACE	1st				2nd
ras1rR1	5′-TCCGTGTGGCGTGTCGAGTTCTATTCTA-3′			ras1rR2	5′-CAGCATACTTCCGGTCGACGGCTAATCT-3′
ras2rR1	5′-CTATGCCTCCGTCTTCGAGGTTCAAGAA-3′			ras1rR2	5′-GTGCCCTGACAATAGATCCGTTCGATCA-3′
ras3rR1	5′-CACCCTCATGCTGGTTGTGACAGTGTTA-3′			ras1rR2	5′-GAGGTTTCCGTCACCGGCCTTCTTGTTA-3′
3′-RACE	1st				2nd
ras1rF1	5′-ATGCTGACGAGGCCTAGAGTAGGCTATA-3′			ras1rF2	5′-CATGCCACTGTCATCGAAGCCAGTTAGT-3′
ras2rF1	5′-CTGCGGTCAGACCAGGGCTAGGTATGCA-3′			ras1rF2	5′-TATGTTCAAGGAGGCGACTGGGGTGCTT-3′
ras3rF1	5′-GACCTAGGGCACACTGTTGGCTCCATCA-3′			ras1rF2	5′-CTGTACCCGCTATCCGAGCACTGGTCTA-3′
(C)					
Ras1	ras1NdeIF		5′-GGAATTCCATATGACCGAGTACAAATTGGTGG-3′		
	ras1BamHIR		5′-CGCGGATCCTCAGCTTAAAAAAGGGTGCAATC-3′		
Ras2	ras2NdeIF		5′-GGAATTCCATATGTCTCGAGCAGGCGACAG-3′		
	ras2BamHIR		5′-CGCGGATCCGGCGTTCGTTACAGGATGGTG-3′		
Ras3	ras3NdeIF		5′-GGAATTCCATATGCGCGAGTACAAAATAGTTG-3′		
	ras3BamHIR		5′-CGCGGATCCCAGCACTCTTACAGAATAATAC-3′		
(D)					
Ras1	ras1iF		5′-GTAGGCGTTGGAAAATCTGC-3′		
	ras1iR		5′-CGCACCCACAACCACCAATT -3′		
Ras2	ras2iF		5′-GTAGGAGTTGGAAAAAGTGC-3′		
	ras2iR		5′-GCCGCCCACCACAACTAACT-3′		
Ras3	ras3iF		5′-GTAGGCGTGGGAAAGTCCGC-3′		
	ras3iR		5′-GCTGCCTAACACAACTATTT -3′		
(E)					
Ras1	ras1qF		5′-GCAACAAGTGCGACCTACAGTCG-3′		
	ras1qR		5′-CGGCTGACTTTATCTTTGCGTATCTC-3′		
Ras2	ras2qF		5′-GCAGGCGACAGACCGAATCAAG-3′		
	ras2qR		5′-TGTCCAAGATGTCCAATTTGGCG-3′		
Ras3	ras3qF		5′-TGGCAAGGAACAGGGACAAAATC-3′		
	ras3qR		5′- GGGGACTTCTTGTTGATCTGTCTCAC-3		

(A) Degenerate primers; (B) Primers for RACE; (C) Primers for full length cDNA constructs; (D) Primers for inverse PCR; (E) Primers for quantitative RT-PCR.

### mRNA Expression Analysis of *Bmras* during Development

**Figure 4:**
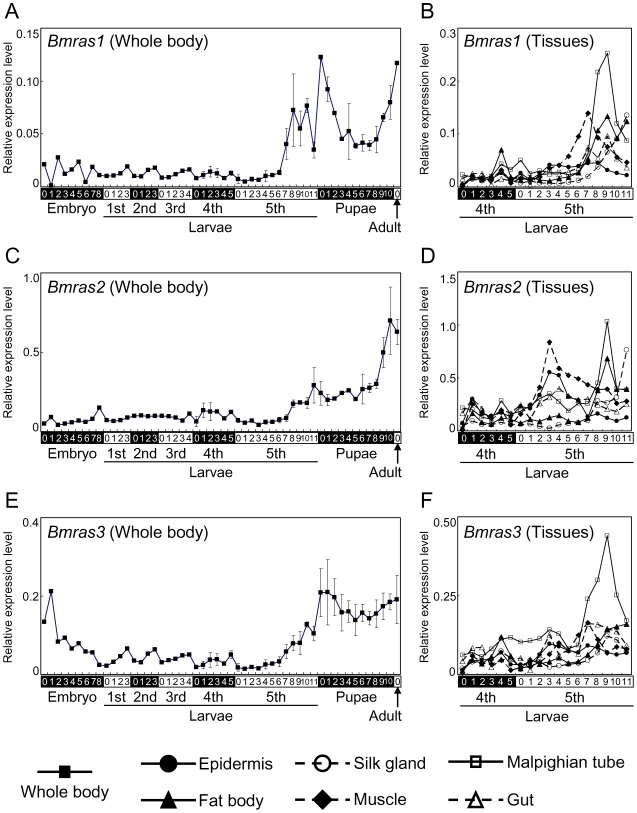
Changes in the mRNA expression levels of *Bmras1*, *Bmras2* and *Bmras3* during development. mRNA samples were harvested from various organs of various developmental stages from the embryo to adult of *B. mori* at 24-hours interval. Transcripts of *Bmras* in these samples were quantified by qRT-PCR. Relative expression levels in whole body (A) and tissues (B) of *Bmras1*, whole body (C) and tissues (D) of *Bmras2*, whole body (E) and tissues (F) of *Bmras3* against *Bmrp49* are shown. Expression levels in whole body samples are indicated by solid squares. Changes in organs, namely, the epidermis, fat body, silk gland, muscle, Malpighian tubles and gut are shown by solid circles, solid triangles, open circles, solid diamonds, open squares and open triangles, respectively.

mRNA expression levels of three *Bmras* in various tissues of various developmental stages were measured using the qRT-PCR technique (Fig. 4). In whole body samples, *Bmras2* (Fig. 4C) transcripts tended to be more abundant than *Bmras1* (Fig. 4A) and *BmRas3* (Fig. 4E) through the development. All *ras* transcripts were low level until the 4th instar, and gradually increased from day 6 of the 5th instar. They then showed a high expression in the pupal stage. However, when expression patterns of *Bmras* were compared precisely, there were differences among their expression patterns. *Bmras1* and *Bmras3* initially had an intensive expression, a moderate expression in the middle and a high expression again at the end of the pupal stage. In addition, they have small peaks of expression at the end of each larval stadium. On the other hand, *Bmras2* were not highly expressed until the end of the pupal stage, with no peaks at the end of each larval stadium except for the one of the 5th stadium. *Bmras3* was also expressed intensely in the early days of the embryonic stage. All *Bmras* were ubiquitously expressed in all tissues used in this study (Fig. 4B, D and F). They did not show obvious patterns of changes in their expression levels except for several peaks. In the Malpighian tube on day 9 of the 5th instar, all *Bmras* showed a clear peak of expression. *Bmras1* and *Bmras2* also showed a peak in the muscle on day 7 and day 3 of the 5th instar, respectively.

### Localization of BmRas-GFP Fusion Proteins in Sf-9 Cells

**Figure 5:**
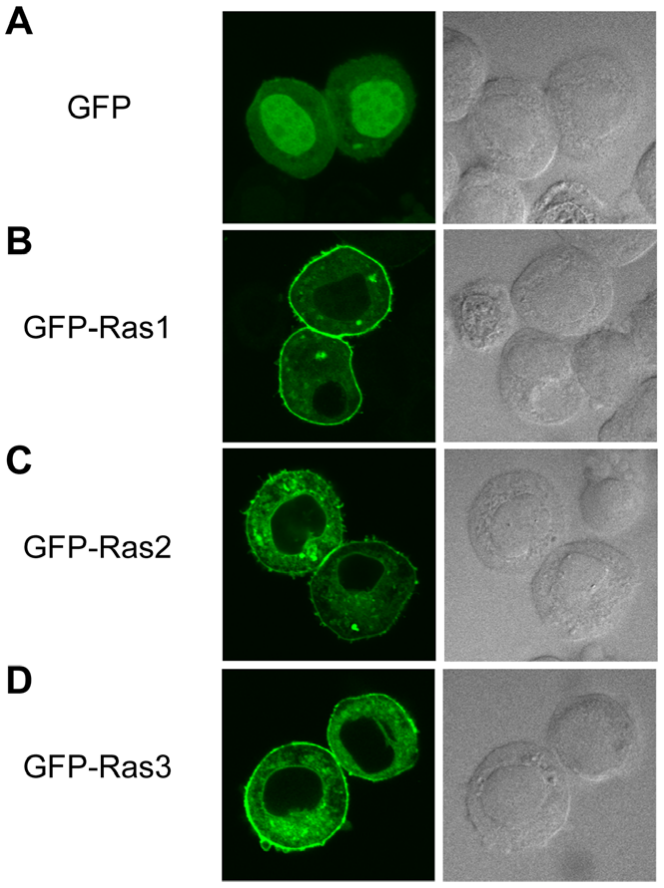
Localization of Ras-GFP fusion proteins in Sf-9 cells. GFP (A), Ras1-GFP (B), Ras2-GFP (C) and Ras3-GFP (D) proteins were translated in Sf-9 cultured cells, and the localization of these proteins was observed with their GFP signals by confocal microscopy (left pannels). Right panels show Nomarsky microscope images of left panels. GFP luminescence is localized in nuclei in the case of non-fused GFP (A), but on the plasma membrane, and weakly in the cytoplasm, in cases of fusion proteins (B, C and D).

Genes coding the constitutively active-type of mutant of three BmRas, which were fused with GFP, were transiently introduced into Sf-9 cells and the cellular localization of their products was observed. Non-fused GFP prepared as a control was widely distributed throughout the cell, although relatively high luminescence was observed in the nuclei (Fig. 5A). In contrast, three BmRas-GFP proteins were specifically localized on the cell membrane (Fig. 5B, C and D), as in the case of mammalian Ras [60]. The weak GFP fluorescence observed in the cytoplasm is thought to be the result of products being transported to the membrane.

## Discussion

### Sequence Analysis of Ras Family Members

Ras small GTPase is one of the most intensively studied molecules in signal transduction. However, its functions have not been investigated in insects except for *D. melanogaster*. Here, we try to clarify the function of Ras in *B. mori*, a model insect of endocrinological studies, to understand its roles in the hormonal regulation of insect development. From the sequence homology of Ras proteins, we cloned *ras* cDNAs from *B. mori* and determined their sequences. As in the case of *D. melanogaster* [40]–[42], three *ras* genes were identified (Fig. 1). Phylogenetic analysis revealed that three Ras of *B. mori* corresponded to those of *Drosophila* (Fig. 3). There were some differences in amino acid residues at effector interaction sites, GEF interaction sites and the C terminal CaaX motif among the subfamily of Ras. Such differences classified three BmRas into separate subgroups. In phylogenetic analysis, only BmRas1 was grouped into the ‘authentic’ Ras subfamily with DmRas1. On the other hand, BmRas2 and BmRas3 were classified into mammalian R-Ras and Rap subfamily with DmRas2 and DmRas3, respectively, and it was consistent with previous reports [8], [61]. In some insect species, sequences which are highly homologous to BmRas1 have been predicted from genome data as Ras, and BmRas2 and BmRas3 as ‘Ras-like’ proteins in the database (http://blast.ncbi.nlm.nih.gov/Blast.cgi). Those ‘Ras’ proteins were also clustered with ‘authentic’ Ras (Fig. 3), and ‘Ras-like’ proteins were with R-Ras or Rap. Therefore, it is likely that many insects possess counterparts of mammalian ‘authentic’ Ras, R-Ras and Rap. Both DmRas2 and mammalian R-Ras were reported to be involved in the formation of the neuronal network [62], [63], suggesting that BmRas2 have similar roles. Recent studies including the genome database of *D. melanogaster* revealed that DmRas3 is identical to *Drosophila* Rap1 (roughened) [48], [64]. *Drosophila* Rap1 was shown to relate to the regulation of cell shapes by forming adherens junctions [65], [66], as in the mammalian Rap subgroup [67]. Therefore, BmRas3 (Rap) might also act in adherens junction formation. In comparison with the Ras subgroup, functions of R-Ras and Rap subgroups have not been elucidated adequately. Further study of insect Ras2 and Ras3 might bring the basis of functions of these subgroups.

### Gene Expression Analysis of *Bmras*

In qRT-PCR analysis, transcripts of three *B. mori* Ras showed ubiquitous expressions, although their expression patterns were different each other. Therefore, it is predicted that three BmRas have different roles, and all are important for the development of *B. mori*. Their ubiquitous expression is consistent with *D. melanogaster* [68], [69]. However, *ras2*, which was the most abundant in *B. mori* (Fig. 4), was less abundant than *ras1* and *ras3* in *D. melanogaster*. Thus, functions of these Ras proteins might be different among insect species. In this study, mRNA expression patterns of BmRas1 and BmRas3 were similar, and it was consistent with previous reports which indicated the related functions of Ras and Rap of *D. melanogaster* [70], [71]. The spatial expression patterns of BmRas were also ubiquitous, indicating their widespread roles in *B. mori*. In *Drosohila*, Ras transcripts were markedly expressed in imaginal discs [69]. It is consistent with the intensive expression of BmRas transcripts after the late stage of 5th instar, the period of adult tissue development. However, *Bmras* were also expressed in the silk gland (a larval tissue) in this study. Thus, it is probable that BmRas exists generally in various tissues, but acts via different machineries in each organ.

The peak of expression in the Malpighian tube in 5th instar larvae on day 9, which was observed for all three BmRas, was interesting. In *Drosophila*, it was shown that Ras controls the development of the Malpighian tube via EGFR in the embryonic period [72]. However, there have been no reports of changes occurring in Malpighian tube in larval stages. It might be related to imaginal disc growth in the puapl stage or signal regulation to excrete endogenous contents unnecessary for pupal metamorphosis. Further investigation is required to elucidate the meaning of Ras signaling activation at the Malpighian tube.

In this study, the expressions of three *Bmras* in the larval stage except for the late 5th stadium were low in comparison with embryonic and pupal stages. This period overlaps with that during which JH exists in a larval body, suggesting involvement of JH in suppression of *ras* expression. Therefore, the signaling of the control of cell differentiation by Ras proteins might be restricted by JH during growth period with limited cell differentiation in the larval stage, and then released in pupal stage with rapid re-formation of body structures which is undergone through determination of the fate of various cells such as apoptosis. Elucidation of the function of JH for *Bmras* expression in specific tissues or cells is predicted to reveal the molecular action of JH and factors related to the action.

### Membrane Localization of BmRas-GFP Fusion Proteins

Mammalian H-, K- and N-Ras have separate tissue-specific mRNA expression patterns [25]. Furthermore, these three Ras are targets of different modification of C terminal, namely isoprenylation and palmitoylation [73]. Recent studies indicated that such tissue specificities and differences of the localization, which is controlled by C terminal modification, cause the differences in functions of mammalian three Ras [60] suggesting the C terminal modification in insects also plays an important role. We showed the membrane localization of three BmRas using GFP-fusion proteins (Fig. 5), which indicated that these proteins had modified C terminals to act in membrane signal transduction as generally seen with Ras proteins. It is predicted that further study of C terminal modification will reveal the molecular mechanism of Ras function in insects.

A recent study of *D. melanogaster* mutants, which have abnormal Ras/MAPK and other signaling pathways, showed that Ras and its downstream components, Raf and PI3K (phosphoinositide-3-kinase), controls the body size of the fly, by regulating the timing of secretion of ecdysone from the prothoracic gland (PG) [56]. Ecdysone is synthesized in PG by the stimulation of the prothoracicotropic hormone (PTTH). Although the molecular mode of action of PTTH is not very clear, complex interactions of kinase pathways, for example protein kinase A, protein kinase C (PKC) and tyrosine kinase pathways, were suggested to be involved in the stimulation of PG by PTTH in *Manduca sexta*, via Ca^2+^ and/or cAMP as second messengers [74]. Therefore, it is predicted that the Ras/MAPK signaling pathway strongly interacts with those signaling pathways to control the ecdysteroidogenesis. Further study for understanding the entire network of ecdysteroidogenesis induced by PTTH via Ras and the interaction of JH is expected to elucidate the framework of the hormonal regulation of the development of insects. BmRas-GFP proteins are beneficial to investigate the molecular interaction among Ras, components of protein kinase pathways and factors related to the hormonal control.

In conclusion, we isolated and identified three genes of Ras family members of *B. mori*, BmRas1, BmRas2 and BmRas3, which corresponded to DmRas1, Ras2 and Ras3, respectively, and showed high sequence homology with their counterparts. mRNA expression analysis revealed that three BmRas are ubiquitously expressed, although they have remarkable expression in late 5th instar and pupal periods with different patterns each other. In addition, all three *Bmras* have a peak of expression in the Malpighian tube in the 5th instar on day 9. Furthermore, we showed the membrane localization of BmRas using a transient expression technique in insect cultured cells as GFP-fusion proteins. These data suggest that three BmRas have characteristics which are common among Ras family members, and thus play central roles in the cell differentiation and proliferation of *B. mori*. Further study will elucidate not only the relationship between the Ras/MAPK pathway and the development of *B. mori*, but also new components and the precise framework of the Ras-related signaling pathway.

## Materials and Methods

### RNA Isolation

*B. mori*, C145xN140 were routinely reared on an artificial diet at 26°C under a 12 hr light/12 hr dark photoperiod. Under these condition, durations of embryo, 1st, 2nd, 3rd, 4th and 5th instars and the pupal stage were 9, 4, 4, 5, 6, 12 and 11 days, respectively. For whole body samples, larvae were dissected and gut contents were removed, and then the whole insect bodies were used as samples. For tissue samples, insects were dissected and the epidermis, fat body, gut, muscle, silk gland and Malpighian tubule were separated. Total RNA was extracted from whole insect bodies from each day (24 hr period) in every stage and from tissues on all days of 4th and 5th instar larvae (24 hr period) using ISOGEN (NIPPON GENE Co., Tokyo, Japan), and purified by SV Total RNA Isolation System (Promega Co., Madison, WI, USA), which includes DNase treatment in its protocol. Total RNA was also extracted from whole insect bodies on the first day of the adult stage and pupal follicle cells. The numbers of insects used for RNA isolation were 40, 10, 5, 2–3, 1, 1, 1 and 1/day for the embryo stage, the larval stadium (1st, 2nd, 3rd, 4th, and 5th), the pupal stage and adult stage, respectively. Concentrations of RNA samples were determined using an UV spectrophotometer NanoDrop ND1000 (Thermo Fisher Scientific Inc., Waltham, MA, USA).

### cDNA Cloning

All primers used in this study were purchased from Tsukuba Oligo Service Co. (Ibaraki, Japan). First-strand cDNAs were synthesized from a total RNA sample of fat bodies of 5th instar day 3 larvae using Ready-To-Go RT-PCR Beads (GE Healthcare, Chalfont St Giles, UK) with oligo(dT) primers. The total RNA sample was treated with DNase (Promega), and 2 µg of the sample was used for cDNA synthesis. From amino acid sequence homologies among Ras proteins, degenerate primers were designed (Table 1). Reverse transcription PCR was conducted with primers ras1-F1 and -R1 for *Bmras1*, ras2-F1 and -R1 for *Bmras2* and ras3-F1 and -R1 for *Bmras3*, respectively. The PCR program was as follows: initial incubation (95°C; 2 min) was followed by 35 repetitions of PCR cycle (95°C; 30 sec, 48°C; 30 sec, 72°C; 2 min). PCR products were then purified and their sequences were determined by ABI PRISM 3100 Genetic Analyzer (Applied Biosystems Co., Foster City, CA, USA) using BigDye Terminator v3.1 Cycle Sequencing Kit (Applied Biosystems). For RACE, 2 µg of total RNA was used for first-strand cDNAs synthesis using SMART RACE cDNA Amplification Kit (TAKARA BIO Inc., Shiga Japan) according to the manufacture's instruction. The primary amplifications were performed by touchdown PCR using proof-reading KOD-PLUS (TOYOBO Co., Osaka, Japan) with primers ras1-rF1 and -rR1 for *Bmras1*, ras2-rF1 and -rR1 for *Bmras2* and ras3-rF1 and -3rR1 for *Bmras3*, respectively (Table 1). PCR products were then further amplified using KOD-PLUS and nested primers, ras1-rF2 and -rR2 for *Bmras1*, ras2-rF2 and -rR2 for *Bmras2* and ras3-rF2 and -rR2 for *Bmras3*, respectively (Table 1). DNA sequences of amplified fragments were determined as mentioned above.

Homological alignment was investigated using CLUSTALW (http://align.genome.jp/). The phylogenetic tree was constructed with UPGMA methods.

### Quantitative Real-Time PCR (qRT-PCR)

Isolated total RNA was reverse transcribed using ReverTra -*Plus*- (TOYOBO) following the manufacturer's instruction. Briefly, 200 ng total RNAs was converted to cDNA with Oligo(dT)_20_ by Moloney murine leukemia virus reverse transcriptase in 20 µl reaction volume. The reaction was diluted to 1/4 concentration with water and used as a template of qRT-PCR. cDNAs encoding full-length open-reading frames (ORFs) of BmRas1, BmRas2 and BmRas3 were amplified by PCR using primer pairs ras1-NdeIF and -BamHIR, ras2-NdeIF and -BamHIR and ras3-NdeIF and -BamHIR, respectively (Table 1), and ligated into pET-16b plasmid vector (Merck KGaA, Darmstadt, Germany). Serial dilutions of these constructs, namely pET16/BmRas1, pET16/BmRas2 and pET16/BmRas3, were used as standards. As a reference gene, *B. mori* ribosomal protein 49 (*Bmrp49*) was chosen. The qRT-PCR standards and primers for *Bmrp49* were prepared as described previously [75]. qRT-PCR primer pairs for *Bmras1*, *Bmras2* and *Bmras3* are listed in Table 1. The transcripts of *Bmras*, the same as *Bmrp49*, in prepared cDNA templates were quantified on a real-time thermal cycler, LightCycler 480 Real-Time PCR System (Roche Diagnostics, Indianapolis, IN, USA). qRT-PCR was carried out in 20 µl reaction volume containing 2 µl template cDNA (equivalent to 5 ng total RNA) or standard cDNA, 1 x SYBR Premix EX Taq (TaKaRa) and each primer. We used a PCR protocol of 1 cycle of denature at 95°C for 10 seconds; 20°C/second followed by 50 cycles of PCR at 95°C for 5 seconds; 20°C/second, 60°C for 20 second; 20°C/second. After the PCR stage, the absence of unwanted by-products was confirmed by automated melting curve analysis. The molar amounts of transcripts of each gene were calculated based on crossing point analysis, with standard curves generated from standard cDNA. The molar amounts of *Bmras* were normalized with *Bmrp49* transcript levels in the same cDNA templates and used as indicators of the expression levels of each gene.

### Expression of BmRas-GFP Fusion Proteins in Sf-9 Cultured Cells

Protein expression constructs of constitutively active mutants of three *Bombyx* Ras as GFP fusion proteins, namely BmRas1Val^12^GFP, BmRas2Val^22^GFP and BmRas3Val^12^GFP, were prepared as follows, with refeence to a previous report [76], [77]. A DNA fragment which codes GFP is introduced into the cloning site of pIB/V5-His-DEST plasmid vector (Invitrogen Co., Carlsbad, CA, USA). Inverse PCR was conducted to amplify mutated *Bmras*, in which a glycine residue at the corresponding position is substituted by a valine residue, using primers ras1-iF and -iR for BmRas1Val^12^GFP, ras2-iF and -iR for BmRas2Val^22^GFP and ras3-iF and -iR for BmRas3Val^12^GFP (Table 1). Templates of inverse PCR were pET16/BmRas1, pET16/BmRas2 and pET16/BmRas3, respectively. Then amplified fragments were inserted into the C-terminal position of the GFP coding site to compose DNA sequences which code Ras-GFP chimeric proteins. These mutant constructs were used for transient transfection to Sf-9 cultured cells, a cell line from lepidopteran *Spodoptera frugiperda*. Sf-9 cells were routinely maintained in Sf-900 II (Invitrogen) at 27°C. For analysis by fluorescence microscopy, 8×10^5^ Sf-9 cells were seeded on the 18-mm micro cover glass (Matsunami Glass Ind., Osaka Japan) in 6-well plates (BD, Franklin Lakes, NJ, USA) and transfected with 1 µg plasmids using Cellfectin (Invitrogen) following the manufacturer's instruction. After 24 hr culture at 27°C, cells were washed three times with 1xPBS (10 mM sodium phosphate, 150 mM sodium chloride, pH = 7.4), fixed with 4% paraformaldehyde for 17 min at room temperature, and washed three times with 1xPBS again. The fixed cells were mounted in Vectashield (Vector Laboratories, Burlingame, CA, USA), and observed using an FV1000 confocal microscope (Olympus, Tokyo, Japan). The images were processed using Photoshop 5.0 (Adobe, San Jose, CA, Japan).
